# Microbiological and Clinical Aspects of *Raoultella* spp.

**DOI:** 10.3389/fpubh.2021.686789

**Published:** 2021-08-02

**Authors:** Tobias M. Appel, Natalia Quijano-Martínez, Elsa De La Cadena, María F. Mojica, María Virginia Villegas

**Affiliations:** ^1^Klinik für Innere Medizin II, Universitätsklinikum Jena, Jena, Germany; ^2^Grupo de Resistencia Antimicrobiana y Epidemiología Hospitalaria, Universidad El Bosque, Bogotá, Colombia; ^3^Facultad de Medicina, Universidad De Los Andes, Bogotá, Colombia

**Keywords:** *Enterobacteriaceae*, MALDI-TOF, *Raoultella planticola*, *Raoultella ornithinolytica*, *Raoultella terrigena*, *Raoultella electrica*

## Abstract

The genus *Raoultella* was established in 2001. Species of *Raoultella* and *Klebsiella* share many ecological, biochemical, clinical, and microbiological features. Given the shortcomings of available technology for species identification in the clinical microbiology laboratory, are practically indistinguishable. Since the late 2000s there has been an increase in case reports of human *Raoultella* infections. Therefore, several authors are postulating that *Raoultella* spp. are rare and/or emerging pathogens.

**Conclusions:***Raoultella* spp. are very similar to *Klebsiella spp*. The epidemiology and the clinical relevance of the human *Raoultella* spp. infections is uncertain and further studies are required. The previous difficulties in the identification of *Raoultella* spp. and the introduction of more precise identification techniques may explain the recent increase in the number of case reports. *Raoultella* spp. might be rather underdiagnosed than rare or emerging pathogens.

## Introduction

Since the late 2000s, the number of case reports of human *Raoultella*-infections available in the literature has been growing remarkably (1). From the 1980s until the 2006, only four case reports of human *Raoultella*-infections are available on PubMed, in contrast to more than 130 today. Due to this recent increase, several authors have been suggesting that *Raoultella* spp. are rare and emerging pathogens.

Herein, we review the literature regarding microbiological and clinical aspects of *Raoultella* spp. and compare key points of this genus with *Klebsiella pneumoniae* and *K. oxytoca*, two common human pathogens in the community, as well as in healthcare settings.

## Methods

We reviewed the literature available as full text in English on PubMed until June 2021, using the search terms “Raoultella,” “Raoultella planticola,” “Raoultella ornithinolytica,” “Raoultella terrigena,” “Klebsiella trevesanii.” All clinical case reports, case series, retro- and prospective studies reporting *Raoutella*-infections, regardless of the microbiological diagnostic approach were included.

## Microbiological Aspects

The *Enterobacteriaceae, Klebsiella planticola*, and *K. terrigena* were first described in 1981, while *K. ornithinolytica* was described 8 years later (2–5). *K. trevisanii* was first described in 1983 but turned out to be identical with *K. planticola* (3). In the late 1990s, modern taxonomic assays such as DNA sequencing of housekeeping genes catalyzed a variety of modifications in bacterial taxonomy (6). In 2001, based on the molecular analysis of 16S ribosomal RNA (rRNA) and RNA polymerase β subunit encoding genes (*rpoB*), *K. planticola, K. ornithinolytica*, and *K. terrigena* were classified into a new genus. In honor to Didier Raoult it was named *Raoultella* (3). Thirteen years later, a novel specie was discovered and named *R. electrica*, which has not been described in human infections (7).

As well as *Klebsiella* spp., *Raoultella* spp. are ubiquitous in nature, being found in plants, water and soil, and are known to colonize humans and animals (1). Both genera are facultative anaerobe Gram-negative bacilli, which belong to the family of *Enterobacteriaceae* and tend to form large mucoid colonies on McConkey agar, due to synthesis of a polysaccharide capsule. Therefore, both genera are morphologically indistinguishable and share several biochemical characteristics, such as the production of catalase, the absence of oxidase, the fermentation of glucose, lactose, sorbose and the reduction of nitrates (8). However, there are a some metabolic features which are present in some species, but not in others, and therefore are useful for their distinction, such as the indole-test, growth at 10°C, the production of histamine, D-melezitose test and the metabolism of ornithine (9) (Table 1), as well as pectate degradation, pigment production on gluconate-ferric citrate agar, and the utilization of gentisate in hydroxybenzoate as a sole carbon source (10, 11). These physiological and biochemical properties can be used for microbiological diagnosis, which are the basis of analytical profile index (API) based systems.

**Table 1:** Metabolic characteristics of clinically relevant species of *Raoultella, K. pneumoniae* and *K. oxytoca*.

**Microorganism**	**Metabolic characteristic**				
	**Indole**	**Growth at 10^**°**^C**	**Histamine production**	**D-melezitose**	**Ornithine metabolism**
*K. pneumoniae*	–	–	–	–	–
*K. oxytoca*	+	+	–	–/+	–
*R. planticola*	–/+	+	+	–	–
*R. terrigena*	–	+	+	+	–
*R. ornithinolytica*	–	+	+	–	+

*(+) positive reaction; (–) negative reaction; (–/+) variable. Table adapted from Alves et al. (9)*.

Several virulence factors such as hemagglutinins, type 1 and type 3 fimbriae, siderophores, enterobactin and occasionally aerobactin are shared between *R. planticola, R. terrigena*, and *K. pneumoniae* (12, 13). Podschun et al. observed that type 1 fimbriae, which are one of the most important proteins for tissue adhesion in *Klebsiella* spp., are less frequently expressed in *R. terrigena*. Exopolysaccharide production and biofilm formation was observed in *R. planticola* and the expression of bacteriocins were described in *R. ornithinolytica* (14, 15). In *R. terrigena*, the production of tetrodotoxin, the potent neurotoxin of *Takifugu niphobles* (commonly known as puffer fish), has been described (16). This is consistent with the hypothesis that tetrodotoxin is not produced by fish. Instead, it might be ingested and accumulated by fish through the food chain, since several tetrodotoxin containing species of fish have been discovered (17).

Species from both genera are intrinsically resistant to penicillins because of the expression of a Ambler class A β-lactamase: LEN-1 or SHV-1 in *K. pneumoniae*, K1 in *K. oxytoca*, and PLA-1, ORN-1, and TER-1 in *R. planticola, R. ornithinolytica*, and *R. terrigena*, respectively (18, 19). A study by Walckenaer et al. demonstrated that PLA-1 and ORN-1 β-lactamases are chromosomally encoded and are 94% identical in their 291 amino acid sequence. This unusual high homology might be a result of an ancestral horizontal gene transfer. Simultaneously, they share a homology of 66–69% with the amino acid sequence of the chromosomal β-lactamases SHV-1/LEN-1 and 69–71% with the plasmid-mediated TEM-1 (18). TER-1, the β-lactamase described in *R. terrigena* shares a 78% homology with the amino acid sequence of the β-lactamases of *R. planticola* and *R. ornithinolytica*, 69.9% with TEM-1 and 67.5% with SHV-1/LEN-1 (19). PLA-1, ORN-1, and TER-1, as well as LEN-1 can be inhibited *in vitro* with β-lactam β-lactamase inhibitors, such as clavulanate, sulbactam, and tazobactam (18, 19).

### Challenging Microbiological Diagnosis

Although the increase in frequency of reported *Raoultella* spp. isolates is remarkable, the microbiological diagnosis is difficult. The reliable identification is challenging due to the close phylogenetic relationship with *Klebsiella* spp. (1). For instance, Mori et al. identified among 439 clinical isolates of *Klebsiella spp*. 18.5% of [*R*.] *planticola* using biochemical and physiological methods (10).

Automated identification systems are commonly used in clinical microbiology laboratories. Three studies available in the literature evaluated the diagnostic performance in identifying species of *Raoultella* of these systems: Park et al. reported that only Vitek 2 system (bioMérieux, Marcyl'Etoile, France) was able to identify correctly all 27 *R. ornithinolytica* isolates, whereas the MicroScan Neg Combo 32 panel (Beckman Coulter, CA, USA) and API 20E (BioMerieux, Marcy-l'Etoile, France) identified 25 and 24 isolates, respectively, as *K. oxytoca* (20). Furthermore, Ponce-Alonso et al. found that MicroScan WalkAway and API 20E failed to detect all 11 *Raoultella* spp. and a study by De Jong reports the misidentification of 3 out of 3 *R. ornithinolytica* and 2 of 2 *R. planticola* isolates as *K. oxytoca* by BD Phoenix (BD, Sparks, USA) (21, 22). In a case report, Demiray et al. described a clinical isolate, which initially was identified as *R. planticola* by Vitek 2. However, 16S rRNA encoding gene analysis resulted in the identification as *R. terrigena* (23).

To date, MicroScan (24) and BD Phoenix systems (25) are able to identify *R. ornithinolytica*, but not *R. planticola* or *R. terrigena*. The API 20E system is able to identify *R. ornithinolytica* and *R. terrigena*, but requires additional tests for the identification of *R. planticola* (26). This data strongly suggests that most of the automated identification systems commonly used in clinical microbiology laboratories fail to reliably identify *Raoultella* spp., thus, the prevalence of this genus in clinical isolates might be underestimated.

The broad introduction of matrix-assisted laser desorption ionization–time of flight mass spectrometry (MALDI-TOF MS) for the identification of microorganisms in hospital scenarios took place in the late 2000s might have further driven the correct identification of *R. ornithinolytica* and *R. planticola* case reports as mentioned by Seng et al. (27). Nevertheless, there are reports of the misidentification of *Raoultella* spp. with MALDI-TOF MS, although only at the species level (22, 28).

Molecular studies are the gold-standard for microbiological identification. Currently, these methods are not routinely used in clinical laboratories for microbiological identification. Sequencing of phylogenetically preserved sequences has been the technique most frequently used. In *Raoultella* spp. and other Enterobacterales *rpoB* and the 16S ribosomal RNA encoding gene are analyzed frequently. In addition, the identification is specific variants of *gyrA* and *parC*, as well as the presence of *bla*_ORN_, *bla*_PLA_, and *bla*_TER_ genes has been described to identify *Raoultella* spp. (19, 29, 30).

Among the 140 case reports of *Raoultella*-infections, only 65% mention the microbiological diagnostic method used (Supplementary Material 1). Of those reporting on the diagnostic method, 61.9% cases of *R. planticola* were diagnosed using the Vitek 2 system (6 of 26 the cases were confirmed with MALDI-TOF MS or molecular analysis), 16.7% using MALDI-TOF. MicroScan systems were used in 48.6% of clinical cases for microbiological diagnosis *of R. ornithinolytica*, followed by Vitek 2 (24.3%) and MALDI-TOF (18.9%). Regarding *R. terrigena*, API 20E was used in three out of eight cases.

## Clinical Aspects of *Raoultella*-Infections

### Epidemiology and Clinical Manifestations

The epidemiology of *Raoultella-*infections is uncertain since only a few surveillance studies are available in the literature and due to the difficulties with automated diagnostic platforms widely used in clinical microbiology laboratories, the incidence might be underestimated. Chun et al. reported *R. planticola* and *R. ornithinolytica* to be isolated in ~0.3 and 0.15% of bacteremia cases, respectively, in a hospital in South Korea. Of note, in about one third of blood samples in which *Raoultella* spp. were isolated, polymicrobial cultures were obtained (31, 32). In a prospective study from Ethiopia, *R. terrigena* was isolated in the urine of 8.38% of 429 participants (8.1% in women, 9.1% in men) with urinary tract infections (33).

Since the first description of *Raoultella* spp., 76 of case reports of *R. planticola*-, 55 of *R. ornithinolytica-* and 9 of *R. terrigena-*infections have been reported (Supplementary Material 1). The recent increase in case reports of *Raoultella*-infections over the last decade has led some authors to the conclusion that *R. planticola* and *R. ornithinolytica* are emerging pathogens (27, 34–36).

In contrast to the case reports, there are 22 studies available on PubMed, which identified 481 isolates of *R. planticola*, 566 of *R. ornithinolytica* and 168 of *R. terrigena* between 1984 and 2015 in several countries in Europe, Asia, Africa and South America (Supplementary Material 1). Most studies evaluated the incidence of isolates of only one *Raoultella* species, three studies focused specifically on bacteremia, two on urinary tract infections and bacteriuria, one on pneumonia and one exclusively on bile-tract infections.

Regarding colonization, *R. planticola* was found in 9.9% of the gut microbiome and upper respiratory tract of 131 neonates in a study from Germany (8), while *R. terr*í*gen*a was isolated in feces in 0.9% of 5,377 healthy adults (37). *R. ornithinolytica* has been identified in human saliva (38, 39), as well as in stool samples of healthy participants in rural Chinese communities (40).

Like *Klebsiella* spp. and other Enterobacterales, species of *Raoultella* can cause a broad range of clinical syndromes. There are only few surveillance studies, all are single-center studies, two of them focusing exclusively on bloodstream infections. Boattini et al. reported 30 infections caused by *R. planticola* and 27 by *R. ornithinolytica* in a hospital in Portugal between 2010 and 2014 (41). In this study, 29 patients had urinary tract infections, nine pneumonia and five bacteremia. Patient's age ranged from 30 to 99 years (average of 68.9 years) in patients with *R. planticola* infections and from 28 to 90 years (average of 66.1 years) in the *R. ornithinolytica* group. Immunodeficiency was reported in 56.3% of patients, such as diabetes (28.1%), malignancy (17.5%), or recipients of a solid organ transplant (14%).

Similarly, a study by Seng et al. reported a series of 112 patients between 1 and 99 years (average of 66 years) with *R. ornithinolytica*-infections mainly of the urinary tract (32%), respiratory tract (24%) and the bloodstream (5%) (27). Twenty-five percent of the 112 patients were classified as immunodeficient, of these, 25% had a diagnosis of solid cancer and 22% had diabetes.

Among 20 patients with BSI involving *R. planticola*, the average age was 65 years, while the 16 patients with BSI caused by *R. ornithinolytica* were 55.7 years on average (31, 32). Chun et al. reported malignancies in 15 of 16 patients and 17 of 20 patients with *R. ornithinolytica* and *R. planticola* bacteremia, respectively.

In a case series of 11 patients (ten women, one man) with *R. planticola*-pneumonia with an age between 51 and 79 years (average of 70 years), five patients had underlying malignancies, two had chronic obstructive pulmonary disease, two cerebral infarctions or hemorrhage, while one patient had end stage renal disease on hemodialysis. Four patients had polymicrobial cultures. In this case series, consolidation (8 out of 11), ground glass opacities (5/11), pleural effusion (5/11), and micronodules were the most common radiological findings (42).

In a study from Ethiopia, *R. terrigena* was isolated in 8.38% patients (*n* = 429) with urinary tract infections. The majority of patients were between 30 and 45 years of age (24/36) and female (25/36) (33).

In the studies mentioned above, mortality rates ranged from 8% (9/112) in patients with different types of *R. ornithinolytica-*infections, to 43.8% (7/16) in patients with *R. ornithinolytica* bacteremia. Mortality among patients with *R. planticola* infections was reported between 9.1% (1/11) and 15.6% (5/32) (27, 31, 32, 41, 42).

In the reviewed case reports, age of patients ranges from premature newborns to a 97 year-old patient, with an average of 49.7 years. Cases available in the literature report most frequently bloodstream infections, urinary tract, bile tract, and respiratory tract infections, while skin and soft tissue infections, conjunctivitis, and septic arthritis are reported less frequently (Supplementary Material 1). However, this information should be interpreted with caution, since a publication bias of case reports, favoring the publication of more severe and more interesting cases, might not reflect real-life clinical experience. Most patients in the case reports are in a debilitated condition, such as prematurity, advanced age, comorbidities such as diabetes, cancer, as well as requirement of a critical care unit, long-term antimicrobial therapy and chemotherapy or are exposed to endoscopic procedures and catheters. Of the reviewed case reports, 11.4% of patients died (5.4% of patients with *R. ornithinolytica*-infections, 11.8% with *R. planticola*-infections and 44.4% [4/9] with *R. terrigena-*infections), while 8.6% did not report a clinical outcome.

Unlike *Klebsiella* spp., species of *Raoultella* are histamine-producers due to the expression of a histidine-decarboxylase (43, 44). The excess of histamine in ingested fish, especially those of the family scombridae is known as scombroid food poisoning. This condition is associated with exanthema, urticaria, headache, diarrhea, edema, and tachycardia (45). In addition, the production of histamine has been reported to cause red skin flushing in some patients with *Raoultella*-infections (46, 47). Nevertheless, only a few cases reports mention the presence of flushing, while others explicitly report its absence (48).

#### Antimicrobial Therapy

As mentioned above, *Raoultella* spp. are intrinsically resistant to penicillins, due to the expression of a broad-spectrum β-lactamase. Similar to *K. pneumoniae* and *K. oxytoca*, wildtype isolates of *Raoultella* spp. are susceptible to antimicrobials commonly used for the treatment of infections caused by Enterobacterales, such as β-lactams (except penicillins), quinolones, aminoglycosides, tetracyclines, fosfomycin, nitrofurantoin, and polymyxins. Like *K. pneumoniae* and other *Klebsiella* species, a variety of acquired genes have been reported to confer resistance to antimicrobials in environmental and clinical isolates of *Raoultella* spp., including genes encoding for class A, B, and D carbapenemases. Furthermore, the co-expression of different carbapenemases, such as KPC with IMP or NDM, as well as in combination with OXA-48 has been described (35, 49–53). Recently, *R. ornithinolytica* harboring *mcr* genes have been described (54, 55).

There are only few studies on clinical isolates of *Raoultella* spp. available. All studies are from a single center and involved only small numbers of isolates. Seng et al. reported resistance to amoxicillin/clavulanate in 16% of 121 clinical isolates from France, 4% to ceftriaxone, 6% to quinolones, and 10% to trimethoprim (TMP/SMX) (27). The study by Boattini et al. from Portugal reported that all 57 clinical isolates of *Raoultella* spp. were susceptible to cefotaxime, ciprofloxacin and gentamicin. *Three* isolates of *R. ornithinolytica* were resistant to TMP/SMX and cefuroxime, two to nitrofurantoin and one to amoxicillin/clavulanate (41). In the two studies previously mentioned from South Korea, one of the 20 isolates of *R. planticola* was resistant to cephalothin and two isolates were intermediate for cefoxitin. In addition, two isolates were resistant to TMP/SMX and one to gentamicin (31). Among the 16 strains of *R. ornithinolytica*, one isolate was resistant to third and fourth-generation cephalosporins, two were resistant to cefazolin and one was resistant to amoxicillin/clavulanate; two isolates were resistant to TMP/SMX (32).

In a study published in 2020, Sękowska et al. evaluated antimicrobial susceptibility and resistance determinants of 79 clinical isolates of *R. ornithinolytica* and 26 of *R. planticola* collected from 65 patients from a university hospital in Poland. Overall, susceptibility to all tested antimicrobials was high (≥81.9%), except for amoxicillin/clavulanate (9.5% susceptible; 59.1% intermediate). For β-lactams, the highest susceptibility was observed for imipenem (99%), followed by meropenem (98.1%), and cefepime (88.6%). Regarding non-β-lactams, the highest susceptibility was observed for gentamicin (93.3%) and ciprofloxacin (92.4%). Fourteen isolates (nine *R. ornithinolytica* and five *R. planticola*) harbored extended-spectrum β-lactamases, most frequently CTX-M-1 in combination with an unknown variant of TEM. In addition, one strain carried CTX-M-1 plus an unknown variant of VIM. Of note, the high non-susceptibility to amoxicillin/clavulanate (90.5%) in this study is unexpected, since *Raoultella* β-lactamases, as well as most of the ESBLs belonging to CTX-M, TEM and SHV-families are inhibited by clavulanate. In addition, susceptibility to cephalosporins and piperacillin/tazobactam was high (56). This phenomenon could be either related to automated susceptibility testing or the breakpoints applied in the study.

## Discussion

As presented above, the genera *Raoultella* and *Klebsiella* share many ecological, biochemical, clinical, and microbiological features, that given the shortcomings of available technology for species identification in the clinical microbiology laboratory, are indistinguashable in many cases.

The previous difficulties in the identification of *Raoultella* spp. and the introduction of more precise identification techniques in clinical microbiology laboratories might explain the recent increase in the number of case reports. Hence, like other microorganisms that are identified more frequently in recent years, *Raoultella* spp. probably should considered as previously underdiagnosed, rather than rare, novel or emerging pathogens.

Reviewing the cases available in the literature, the (although limited) ability of automated identification systems, as well as the introduction of MALDI-TOF MS in microbiological laboratories are probably the main driver of an increasing identification of this genus. Unfortunately, an important number of case reports are not revealing any information on the microbiological diagnosis. Twenty-two studies available in the literature with 1,215 clinical isolates of *Raoultella* spp. (Supplementary Material 1), suggest that these bacteria probably are not rare pathogens either. Mori et al. described an “unexpectedly high incidence” of *R. planticola* among clinical isolates of *Klebsiella* spp. from Japan, using biochemical and metabolic tests for identification, while a study from Ethiopia reported *R. terrigena* to be isolated in 8.39% of patients with urinary tract infections (10, 33).

Besides the recent increase in case reports of human *Raoultella*-infections, little is known about the real epidemiology of these infections. Only few surveillance studies are available in the literature, all of which are from a single-center and the majority with retrospective design. In addition, some of these studies focused only on the surveillance of a single species of *Raoultella*, while others reported only cases of bloodstream-, urinary tract, respiratory tract or bile tract infections.

Regarding the suggested role of *Raoultella* spp. as opportunistic pathogens, especially in intensive care unit patients, further investigations are required, since in two studies 44 to 75% of patients had no known underlying immunosuppressing condition (27, 41). However, both studies reported an important proportion of polymicrobial infections, enrolled a relatively small numbers of patients, and a homogenous definition of immunosuppression was lacking. On the other hand, there is no standardized and universal definition of “opportunism,” and many species of *Klebsiella* and even some strains of the omnipresent pathogen *K. pneumoniae* are considered as opportunistic pathogens.

The clinical implications of histamine productions by species of *Raoultella* is another relevant feature that requires further investigations. Some case reports mention the presence of a rash which is attributed to histamine production, while other authors explicitly mention its absence (48). High amounts of histamine might have an impact on clinical outcomes and therefore therapeutic implications, especially in blood stream infections and other sever infections in critically ill patients.

Several environmental and clinical isolates of species of *Raoultella* carrying resistance determinants to last-resort antimicrobials, such as carbapenems and colistin have been reported. Some authors hypothesize that *Raoultella* spp. could play a role as a reservoir of antimicrobial resistance genes. Nevertheless, only few surveillance studies on antimicrobial susceptibility in *Raoultella* spp. are available in the literature. Although solid clinical data is lacking, considering microbiological characteristics, the therapeutic approach to patients with *Raoultella*-infections should be similar to patients with infections caused by *Klebsiella* spp., considering individual clinical and pharmacological aspects of the patient, as well as the local epidemiology of antimicrobial resistance.

In conclusion, *Raoultella* spp. are very similar to *Klebsiella* spp. and are probably not rare, neither emerging. Further studies are required to establish the epidemiology and clinical relevance of this genus.

## Author Contributions

TA and NQ-M wrote the first draft of the manuscript. MM and ED contributed very insightful suggestions on microbiological aspects. MV contributed with very insightful suggestions on clinical aspects. All authors contributed to the manuscript revision, read, and approved the submitted version.

## Conflict of Interest

The authors declare that the research was conducted in the absence of any commercial or financial relationships that could be construed as a potential conflict of interest.

## Publisher's Note

All claims expressed in this article are solely those of the authors and do not necessarily represent those of their affiliated organizations, or those of the publisher, the editors and the reviewers. Any product that may be evaluated in this article, or claim that may be made by its manufacturer, is not guaranteed or endorsed by the publisher.
