# Supplementary material for: Microbiological and Clinical Aspects of Raoultella spp
Source: Front Public Health. 2021 Aug 2;9:686789. doi: 10.3389/fpubh.2021.686789 (PMC8365188; doi:10.3389/fpubh.2021.686789)
Supplement: Supplementary file 1 [file Table_1.DOCX]

**Supplemental material**

**Table 1**. Case reports of *Raoultella planticola* available on Pubmed until June 15, 2021

| **Author** | **Year of report** | **Diagnostic tool** | **Sample** | **Patient age** | **Patient sex** | **Region** | **Clinical syndrome** | **Outcome** |
| --- | --- | --- | --- | --- | --- | --- | --- | --- |
| Freney (1) * | 1984 | API 50 gallery | Blood | 47 | M | France | Bacteremia | N/A |
|  |  |  | Blood | 40 | M | France | Bacteremia | N/A |
| Freney (2) * | 1986 | API 20EC gallery, API 147 assimilation gallery, manually | Blood | 69 | F | France | Bacteremia | Clinical cure |
|  |  |  | Blood, Sputum | 57 | N/A | France | Pneumonia | Clinical cure |
| Alves (3) | 2007 | Biochemical testing, *rpoB* sequencing | Peritoneal fluid | 45 | M | Brazil | Pancreatitis | Clinical cure |
| Castanheira (4) | 2009 | 16S rRNA sequencing | Blood | 83 | F | USA | Pneumonia | Death |
|  |  |  | Blood | 64 | M | USA | SSTI | Death |
| O’Connell (5) | 2010 | Vitek 2 (93%) | Pus | 30 | M | Ireland | SSTI | Clinical cure |
| Wolcott (6) | 2010 | PCR (target N/A) | N/A | 66 | M | USA | SSI | N/A |
| Yokota(7) | 2012 | Vitek 2 (>95%) | Blood | 65 | M | Japan | Cholangitis | Clinical cure |
| Kim (8) | 2012 | Vitek 2 (95%), 16S rRNA sequencing | Peritoneal fluid | 66 | M | South Korea | Necrotizing fasciitis | Clinical cure |
| Teo (9) | 2012 | Vitek 2 (99%) | Bile | 62 | F | United Kingdom | Cholecystitis | Clinical cure |
| Lee (10) | 2012 | Vitek 2 (93%) | Blood, Bile | 75 | M | South Korea | Cholangitis | Death |
| Hu (11) | 2012 | N/A | Blood | 59 | M | Canada | Cholangitis | Clinical cure |
| Olson (12) | 2013 | Vitek 2 (93%) | Urine | 89 | M | USA | UTI | Clinical cure |
| Puerta-Fernandez (13) | 2013 | N/A | Blood | 63 | M | Spain | Bacteremia | Clinical cure |
| Tseng (14) | 2014 | Vitek 2, 16S rRNA sequencing | Sputum | 77 | M | Taiwan | Pneumonia | Death |
|  |  |  | Blood | 57 | M | Taiwan | Bacteremia | Clinical cure |
| Koukoulaki (15) | 2014 | Vitek 2 | Urine | 67 | M | Greece | Prostatitis | Clinical cure |
| Nada (16) | 2014 | Vitek 2 (99%) | Pus | 1 | F | Saudi Arabia | SSTI | Clinical cure |
| Lam (17) | 2014 | Vitek 2 | Blood | 56 | F | Canada | Bacteremia | Clinical cure |
| Zuberbuhler (18) | 2014 | N/A | Conjunctival swab | 58 | F | United Kingdom | Conjunctivitis | Clinical cure |
| Salmaggi (19) | 2014 | N/A | Blood | 70 | M | Italy | Cholangitis | Clinical cure |
| Ershadi (20) | 2014 | N/A | Bile | 59 | M | USA | Cholecystitis | Clinical cure |
| Gonzalez-Gonzalez (21) | 2015 | N/A | Blood | 82 | F | Spain | Cholangitis | Clinical cure |
| Xu (22) | 2015 | Vitek 2, 16S rRNA sequencing | Sputum | 60 | M | China | Pneumonia | Death |
| Gangcuangco (23) | 2015 | Vitek 2 | Urine | 92 | M | USA | UTI | Clinical cure |
| Yoon (24) | 2015 | Vitek 2 | Urine | 1 | M | South Korea | UTI (cystitis) | Clinical cure |
| Kim (25) | 2015 | Vitek 2 | Peritoneal fluid | 65 | M | South Korea | Peritonitis | Clinical cure |
| Cho (26) | 2016 | N/A | Sputum | 58 | M | South Korea | Pneumonia | Clinical cure |
| Vassallo (27) | 2016 | Vitek 2 | Conjunctival swab | 88 | F | Malta | Conjunctivitis | Clinical cure |
|  |  |  | Conjunctival swab | 71 | M | Malta | Conjunctivitis | N/A |
|  |  |  | Conjunctival swab | 15 | F | Malta | Conjunctivitis | N/A |
|  |  |  | Conjunctival swab | 69 | F | Malta | Conjunctivitis | Clinical cure |
| Gian (28) | 2016 | N/A | Prostatic fluid | 53 | M | USA | Prostatitis | Clinical cure |
| De Campos (29) | 2016 | Vitek 2 | Peritoneal fluid | 57 | M | Brazil | Peritonitis | Death |
| Sitaula (30) | 2016 | N/A | Blood, urine, pus | 62 | M | USA | Liver abscess | Clinical cure |
| Papaefstathiou (31) | 2016 | N/A | Urine | 42 | M | Greece | UTI | Clinical cure |
| Pan (32) | 2017 | MicroScan WalkAway 40 S1, 16S rRNA sequencing | Sputum, stool | 74 | M | China | Pneumonia | Clinical cure |
| Skelton (33) | 2017 | Vitek 2 (99%) | Urine | 73 | F | USA | UTI | Clinical cure |
| Westerveld (34) | 2017 | N/A | Sputum | 36 | F | USA | Pneumonia | Clinical cure |
| Adjodah (35) | 2017 | Vitek 2 (95%) | Pus | 79 | M | France | CIDI | Clinical cure |
| Tuğcu (36) | 2017 | MALDI-TOF MS (Vitek MS, bioMérieux, Marcy l’Étoile, France) | Urine | 57 | M | Turkey | UTI | Clinical cure |
| Howell (37) | 2017 | N/A | Urine | 1 | F | USA | UTI | Clinical cure |
| Atıcı (38) | 2017 | Vitek 2, MALDI-TOF MS, 16S rRNA sequencing | Conjunctival swab | 0 | F | Turkey | Conjunctivitis | Clinical cure |
| Bonnet (39) | 2017 | MALDI-TOF MS (Brücker) | Synovial fluid | 46 | F | France | Septic arthritis | Clinical cure |
| Bardellini (40) | 2017 | N/A | Oral swab | 16 | M | Italy | Oral infection | Clinical cure |
| Ulukent (41) | 2017 | MALDI-TOF MS (Vitek MS) | Bile | 71 | F | Turkey | Cholecystitis | Clinical cure |
| Subedi (42) | 2017 | N/A | Pus | 63 | M | USA | Osteomyelitis, epidural abscess | Clinical cure |
| Povlow (43) | 2017 | N/A | Blood | 66 | M | USA | Bacteremia | Clinical cure |
| Yoshida (44) | 2017 | N/A | Pus | 74 | M | Japan | Palm atheroma | Clinical Cure |
| Kalaria (45) | 2017 | N/A | Pus | 73 | F | USA | Wound infection | Clinical Cure |
| Yamamoto (46) | 2018 | N/A | Blood | 81 | M | Japan | Bacteremia | N/A |
| Naganathan (47) | 2018 | N/A (“Vitek”) | Blood | 63 | F | Canada | Necrotizing appendicitis | Clinical cure |
| Lam^#^ (48) | 2018 | Vitek 2, MALDI-TOF MS (BioTyper; Bruker Daltonics) | Pus | 85 | M | Canada | Osteomyelitis | Clinical cure |
| Yumoto (49) | 2018 | Vitek 2 | Blood | 79 | M | Japan | Bacteremia | Death |
| Mehmood (50) | 2018 | N/A | Urine | 65 | M | USA | UTI | Clinical cure |
| Gonzales Zamora (51) | 2018 | MALDI-TOF MS | Blood, sputum | 85 | F | USA | Pneumonia | Clinical cure |
| AlSweed (52) | 2018 | N/A | Blood | 4 | M | Saudi Arabia | Bacteremia | N/A |
| Fager (53) | 2019 | N/A | Urine | 51 | F | USA | UTI | Clinical cure |
| Al-Sawaf (54) | 2019 | MALDI-TOF MS (Biotyper, Bruker Daltonics) | Blood | 38 | M | Germany | SSTI | Clinical cure |
| Oliver (55) | 2019 | N/A | Urine | 56 | M | USA | Prostatitis | Clinical cure |
| Pacilli (56) | 2019 | N/A | Peritoneal fluid | 8 | F | Australia | Peritonitis | Clinical cure |
| Park (57) | 2019 | MALDI-TOF MS (Vitek MS, BioMérieux, Durham, North Carolina, USA) | Blood | 35 | M | USA | Bacteremia | Death |
| Asif (58) | 2019 | N/A | Bone | 75 | M | USA | Osteomyelitis | Clinical cure |
| Harmon (59) | 2019 | N/A | Urine | 44 | F | USA | UTI | Clinical cure |
| Tufa (60) | 2020 | MALDI-TOF MS (VITEK MS, bioMérieux, Marcy-l’Étoile, France) | Pus | 17 | F | Ethiopia | SSI | Clinical cure |
| Chen (61) | 2020 | N/A | Blood | 0 | M | China | Bacteremia | Clinical cure |
|  |  |  | Blood | 0 | M | China | Bacteremia | Clinical cure |
|  |  |  | Blood | 0 | F | China | Bacteremia | Clinical cure |
|  |  |  | Blood | 0 | F | China | Bacteremia | Clinical cure |
| Zhao (62) | 2020 | N/A | CSF | N/A | N/A | China | Intracranial infection | N/A |
| Ismair (63) | 2020 | N/A | Blood | 77 | M | USA | Joint infection | N/A |
| Yilmaz (64) | 2021 | Vitek 2 Compact | Peritoneal fluid | 42 | M | Turkey | Peritonitis | Clinical cure |
| Blihard (65) | 2021 | N/A | Blood | 31 | F | Grenada | Cholangitis | Clinical cure |
| Erwes (66) | 2021 | N/A | Blood | 73 | M | USA | Liver abscess, Bacteremia | Death |

(*) Reported as *Klebsiella trevisanii*

# Disconcerting identification: Identified as *R. planticola* by an automatized system and as *R. ornithinolytica* by MALDI-TOF MS. Interestingly, the strain was negative for ornithine carboxylase.

N/A Information not available

M, male

F, female

UTI, urinary tract infection

SSTI, skin and soft tissue infection

SSI, surgical site infection

CIDI, cardiac implantable device infection

**Table 2**. Case reports of *Raoultella ornithinolytica* available on Pubmed until May 10, 2019

| **Author** | **Year of report** | **Diagnostic tool** | **Sample** | **Patient age** | **Patient sex** | **Region** | **Clinical syndrome** | **Outcome** |
| --- | --- | --- | --- | --- | --- | --- | --- | --- |
| Deeb (67) | 2008 | N/A | Blood | 46 | M | USA | Bacteremia | Clinical cure |
| Castanheira (4) | 2009 | 16S rRNA sequencing | Blood | 51 | M | USA | Bacteremia | Death |
| Morais (68) | 2009 | API-20E strip (bioMérieux, Marcy l'Etoile, France) | Blood, Stool | 82 | F | Spain | Enteric fever | Clinical cure |
| Vos (69) | 2009 | N/A | Urine | 97 | F | France | UTI | Clinical cure |
| Mau (70) | 2010 | Vitek 2 (99%) | Blood | 0 | M | USA | Bacteremia | Clinical cure |
| Solak (71) | 2011 | Vitek 2 | Pus | 44 | F | Turkey | SSTI | N/A |
| Sener (72) | 2011 | N/A | BAL | 1 | F | Turkey | Pneumonia | Clinical cure |
| Hadano (73) | 2012 | MicroScan WalkAway 40 plus (Siemens Healthcare Diagnostics, Tokyo, Japan) (99%) | Blood | 92 | M | Japan | Bacteremia | Clinical cure |
|  |  |  | Blood | 52 | F | Japan | Cholangitis, bacteremia | Clinical cure |
|  |  |  | Blood | 59 | M | Japan | Bacteremia | Clinical cure |
| Khajuria (74) | 2013 | Vitek 2 | Pus | 67 | M | India | SSI | Clinical cure |
| García-Lozano (75) | 2013 | MicroScan (Dade Behring de Siemens, Sacramento, CA, USA), API 20E | Urine | 61 | M | Spain | UTI | Clinical cure |
|  |  |  | Urine | 64 | F | Spain | UTI | Clinical cure |
|  |  |  | Urine | 71 | F | Spain | UTI | Clinical cure |
|  |  |  | Urine | 66 | F | Spain | UTI | Clinical cure |
| Sandal (76) | 2014 | N/A | Blood | 0 | F | Turkey | Bacteremia | Death |
| Sibanda (77) | 2014 | N/A | Peritoneal fluid | 53 | M | Botswana | Peritonitis | Clinical cure |
| Haruki (78) | 2014 | Microscan Walkaway 40 SI (Siemens  Healthcare Diagnostics, Tokyo, Japan) | Blood | 73 | F | Japan | Bacteremia | Clinical cure |
|  |  |  | Blood | 75 | M | Japan | Bacteremia | Clinical cure |
|  |  |  | Blood | 92 | F | Japan | Bacteremia | Clinical cure |
|  |  |  | Blood | 44 | M | Japan | Bacteremia | Clinical cure |
|  |  |  | Blood | 65 | M | Japan | Bacteremia | Clinical cure |
|  |  |  | Blood | 77 | F | Japan | Bacteremia | Clinical cure |
| Kaya (79) | 2015 | MALDI-TOF MS (Bruker Daltronics Biotyper), confirmation with biochemical tests | Blood | 37 | M | Turkey | Bacteremia | Death |
| Bhatt (80) | 2015 | Vitek 2 | Drainage fluid | 75 | M | India | Intraabdominal infection | N/A |
| Nakasone (81) | 2015 | N/A | Urine | 73 | F | USA | UTI | Clinical cure |
| Sekowska (82) | 2015 | Vitek 2 Compact (87%), confirmation with MALDI-TOF MS (Bruker Daltronics Biotyper) | Blood | 8 | F | Poland | Bacteremia | Clinical cure |
| Zheng (83) | 2015 | 16S rRNA sequencing | Pus | 13 | M | China | SSI | Clinical cure |
| Yamakawa (84) | 2016 | Microscan Walkaway 40 SI  (Siemens Healthcare Diagnostics, Tokyo, Japan) | Blood | 3 | F | Japan | Bacteremia | Clinical cure |
|  |  |  | Blood | 7 | F | Japan | Bacteremia | Clinical cure |
|  |  |  |  |  |  |  |  |  |
| Venus (85) | 2016 | MALDI-TOF MS (VITEK® MS, bioMérieux Canada Inc.,  St. Laurent, QC, Canada) | Synovial fluid | 68 | F | Canada | Septic arthritis | Clinical cure |
| Seng (86) | 2016 | MALDI-TOF MS, 16S rRNA sequencing | Periprosthetic effusion | 67 | M | France | Prosthetic joint infection | Clinical cure |
| Sueifan (87) | 2016 | N/A | Blood | 57 | M | Germany | Cholangitis, sepsis | Clinical cure |
| Singh (88) | 2017 | Vitek 2 Compact | Pharyngeal swab | 70 | F | India | Sinusitis | Clinical cure |
| Jellinge (89) | 2017 | MALDI-TOF MS (Bruker Daltronics Biotyper) | Sputum | 48 | W | Denmark | Tracheitis | Clinical cure |
| Levorova (90) | 2017 | N/A | Synovial fluid | 38 | F | Czech Republic | Septic arthritis | Clinical cure |
| Abbas (91) | 2018 | Microscan Walkaway 40 SI (Siemens Healthcare Diagnostics, Tokyo,  Japan) | Blood | 0 | M | India | Bacteremia | Clinical cure |
| González-Castro (92) | 2018 | N/A | Blood | 71 | F | Spain | Bacteremia | Clinical cure |
| Papakanderaki (93) | 2018 | N/A | Sputum | 75 | M | Greece | Pneumonia | Clinical cure |
| Lam* (48) | 2018 | Vitek 2, MALDI-TOF MS (BioTyper; Bruker Daltonics) | Pus | 85 | M | Canada | Osteomyelitis | Clinical cure |
| De Petris (94) | 2018 | Microscan Walkaway | Urine | 0 | M | Italy | UTI | Clinical cure |
| Hajjar (95) | 2018 | MALDI-TOF MS | Blood | 54 | M | Canada | Appendicitis | Clinical cure |
| Ayoade (96) | 2018 | MicroScan  Walkway 96 plus | Pus | 24 | W | USA | SSI | Clinical cure |
| Van Cleve (97) | 2018 | N/A | BAL | 39 | M | USA | VAP | Clinical cure |
|  | 2018 |  | BAL | 50 | M | USA | VAP | Clinical cure |
| Büyükcam (98) | 2019 | MALDI-TOF MS (Vitek MS, bioMérieux, Marcy l’Étoile, France) | Urine | 6 | F | Turkey | UTI | Clinical cure |
| Sánchez-Códez (99) | 2019 | N/A | Blood | 11 | M | Spain | Bacteremia | Clinical cure |
| Reyes (100) | 2020 | Vitek 2 | Pus | 64 | M | Ecuador | SSI | Clinical cure |
| Surani (101) | 2020 | N/A | Pus | 84 | M | USA | Liver abscess | Clinical cure |
| Cavaliere (102) | 2020 | MALDI-TOF MS | Pus | 54 | M | Italy | External otitis | Clinical cure |
| Pi (103) | 2020 | Vitek 2 compact | Blood | 0 | M | China | Pneumonia | Clinical cure |
|  |  |  | BAL | 0 | F | China | Pneumonia | Clinical cure |
|  |  |  | BAL | 11 | F | China | Pneumonia | Clinical cure |
|  |  |  | Blood | 11 | M | China | Bacteremia | Clinical cure |
|  |  |  | Blood | 16 | M | China | Bacteremia | Clinical cure |
| Prada-Avella (104) | 2021 | Vitek 2xL | Urine | 9 | M | Colombia | UTI | Clinical cure |

# Disconcerting identification: Identified as *R. planticola* by an automatized system and as *R. ornithinolytica* by MALDI-TOF; interestingly, the strain was negative for ornithine carboxylase.

N/A, information not available

M, male

F, female

UTI, urinary tract infection

SSTI, skin and soft tissue infection

SSI, surgical site infection

CIDI, cardiac implantable device infection

**Table 3**. Case reports of *Raoultella terrigena* available in Pubmed until May 10, 2019

| **Author** | **Year of report** | **Diagnostic tool** | **Sample** | **Patient age** | **Patient sex** | **Region** | **Clinical syndrome** | **Outcome** |
| --- | --- | --- | --- | --- | --- | --- | --- | --- |
| Goegele (105) | 2007 | N/A | Blood | 45 | M | Austria | Endocarditis | Death |
| Shaikh (106) | 2011 | N/A | Blood | 69 | F | United Kingdom | Sepsis | Clinical cure |
| Demiray (107) | 2015 | Identified as R. planticola by Vitek 2, identified as R. terrigena by 16S rRNA sequencing | Urine | 0 | N/A | Turkey | UTI | Clinical cure |
| Wang (108) | 2016 | 16S rRNA sequencing | Pus | 63 | M | China | Subungual abscess | Clinical cure |
| Mal (109) | 2019 | API 20 E | Blood, urine | 30 | F | Pakistan | Sepsis | Death |
|  |  |  | Blood, tracheal aspirate | 36 | F | Pakistan | Pneumonia, Sepsis | Death |
|  |  |  | Urine | 63 | F | Pakistan | UTI | N/A |
| Lekhniuk (110) | 2021 | MIKRO‐LA‐TEST ENTERO kit (ErbaLachema) | Bronchial secretion | 42 | F | Ukraine | Pneumonia | Clinical cure |
|  |  |  | Bronchial secretion | 18 | F | Ukraine | Pneumonia | Death |

N/A, information not available

M, male

F, female

UTI, urinary tract infection

**Table 4**. Studies reporting clinical isolates of *Raoultella* spp.

| **Author** | ***R. planticola*** | ***R. ornithinolytica*** | ***R. terrigena*** | **Region** | **Study period** |
| --- | --- | --- | --- | --- | --- |
| Mori (111) | 81 | 0 | 1 | Japan | 1984-1986 |
| Podschun (112) | ^a^ | ^a^ | 10 | Germany | 1988-1990 |
| Chun (113) | ^a^ | 16^b^ | ^a^ | South Korea | 2002-2011 |
| Seng (114) | ^a^ | 121 | ^a^ | France | 2002-2013 |
| Al-Hulu (115) | ^a^ | 11 | ^a^ | Iraq | 2006-2007 |
| Park (116) | 0 | 27 | 0 | South Korea | 2006-2007 |
| De Jong (117) | 2 | 3 | 0 | Netherlands | 2008-2011 |
| Chun (118) | 20^b^ | ^a^ | ^a^ | South Korea | 2008-2012 |
| Boattini (119) | 32 | 25 | 0 | Portugal | 2010-2014 |
| Ponce-Alonso (120) | 11^b^ | 0 | 0 | Spain | 2011-2014 |
| Demiray (121) | 42 | ^a^ | ^a^ | Turkey | 2011-2015 |
| Hong (122) | 63 (11^d^) | ^a^ | ^a^ | South Korea | 2011-2017 |
| Ahmed (123) | ^a^ | ^a^ | 58 | Pakistan | 2013-2018 |
| Mohammed (124) | 5 | 0 | 27 | Nigeria | 2014 |
| Venkataramanan (125) | 37^e^ | ^a^ | ^a^ | USA | 2015-2020 |
| Seifu (126) | 0 | 0 | 36^e^ | Ethiopia | 2016 |
| Bueno (127) | 69 | 268 | 8 | Spain | 2016-2019 |
| Sękowska (128) | 26 | 79 | 0 | Poland | Not reported |
| Podschun (129) | 92 | ^a^ | ^a^ | Germany | Not reported |
| Podschun (130) | ^a^ | ^a^ | 22 | Germany | Not reported |
| Abid (131) | 1 | 16 | 3 | Iraq | Not reported |
| Arteta (132) | 0 | 0 | 3^c^ | Colombia | Not reported |
| **Total** | **481** | **566** | **168** |  |  |

^a^ Not evaluated or not reported ^b^ Only cases of bacteremia were evaluated ^c^ Only cases of bile tract infections were evaluated. ^d^ Only cases of pneumonia were evaluated. ^e^ Only cases of UTI were evaluated.

**References**

1. Freney J, Fleurette J, Gruer LD, Desmonceaux M, Gavini F, Leclerc H. Klebsiella trevisanii colonisation and septicaemia. Lancet (London, England). 1984;1(8382):909.

2. Freney J, Gavini F, Alexandre H, Madier S, Izard D, Leclerc H, et al. Nosocomial infection and colonization by Klebsiella trevisanii. J Clin Microbiol. 1986;23(5):948–50.

3. Alves MS, Riley LW, Moreira BM. A case of severe pancreatitis complicated by Raoultella planticola infection. J Med Microbiol. 2007;56(5):696–8.

4. Castanheira M, Deshpande LM, DiPersio JR, Kang J, Weinstein MP, Jones and RN. First Descriptions of blaKPC in Raoultella spp. (R. planticola and R. ornithinolytica): Report from the SENTRY Antimicrobial Surveillance Program. J Clin Microbiol. 2009;47(12):4129.

5. O’ Connell K, Kelly J, Niriain U. A Rare Case of Soft-Tissue Infection Caused by Raoultella planticola. Case Rep Med. 2010;2010.

6. Wolcott R, Dowd S. Molecular diagnosis of Raoultella planticola infection of a surgical site. J Wound Care. 2010;19(8):329–32.

7. Yokota K, Gomi H, Miura Y, Sugano K, Morisawa Y. Cholangitis with septic shock caused by Raoultella planticola. J Med Microbiol. 2012;61(Pt 3):446–9.

8. Kim S-H, Roh KH, Yoon YK, Kang DO, Lee DW, Kim MJ, et al. Necrotizing fasciitis involving the chest and abdominal wall caused by Raoultella planticola. BMC Infect Dis. 2012;12:59.

9. Teo I, Wild J, Ray S, Chadwick D. A Rare Case of Cholecystitis Caused by Raoultella planticola [Internet]. Case Reports in Medicine. 2012. Available from: https://www.hindawi.com/journals/crim/2012/601641/

10. Lee JH, Choi WS, Kang SH, Yoon DW, Park DW, Koo JS, et al. A Case of Severe Cholangitis Caused by Raoultella planticola in a Patient with Pancreatic Cancer. Infect Chemother. 2012;44(3):210.

11. Hu AY, Leslie KA, Baskette J, Elsayed S. Raoultella planticola bacteraemia. J Med Microbiol. 2012;61(10):1488–9.

12. Olson DS, Asare K, Lyons M, Hofinger DM. A novel case of Raoultella planticola urinary tract infection. Infection. 2013;41(1):259–61.

13. Puerta-Fernandez S, Miralles-Linares F, Sanchez-Simonet M V, Bernal-Lopez MR, Gomez-Huelgas R. Raoultella planticola bacteraemia secondary to gastroenteritis. Clin Microbiol Infect. 2013;19(5):E236–7.

14. Tseng S-P, Wang J-T, Liang C-Y, Lee P-S, Chen Y-C, Lu P-L. First Report of blaIMP-8 in Raoultella planticola. Antimicrob Agents Chemother. 2014;58(1):593–5.

15. Koukoulaki M, Bakalis A, Kalatzis V, Belesiotou E, Papastamopoulos V, Skoutelis A, et al. Acute prostatitis caused by Raoultella planticola in a renal transplant recipient: a novel case. Transpl Infect Dis. 2014;16(3):461–4.

16. Nada B, Areej M. Raoultella planticola, a central venous line exit site infection. J Taibah Univ Med Sci. 2014;9(2):158–60.

17. Lam PW, Salit IE. Raoultella planticola bacteremia following consumption of seafood. Can J Infect Dis Med Microbiol. 2014;25(4):e83–4.

18. Zuberbuhler B, Abedin A, Roudsari A. A novel case of chronic conjunctivitis in a 58-year-old woman caused by Raoultella. Infection. 2014;42(5):927–9.

19. Salmaggi C, Ancona F, Olivetti J, Pagliula G, Ramirez GA. Raoultella planticola-associated cholangitis and sepsis: a case report and literature review. QJM An Int J Med. 2014;107(11):911–3.

20. Ershadi A, Weiss E, Verduzco E, Chia D, Sadigh M. Emerging pathogen: a case and review of Raoultella planticola. Infection. 2014;42(6):1043–6.

21. González-González L, Álvarez-Otero J, Lamas Ferreiro JL, de la Fuente Aguado J. Colangitis y bacteriemia por Raoultella planticola. Med Clin (Barc). 2015;144(5):231–2.

22. Xu M, Xie W, Fu Y, Zhou H, Zhou J. Nosocomial pneumonia caused by carbapenem-resistant Raoultella planticola: a case report and literature review. Infection. 2015;43(2):245–8.

23. Gangcuangco LMA, Saul ZK. A novel case of Raoultella planticola urinary tract infection in a female: comment on ‘Nosocomial pneumonia caused by carbapenem-resistant Raoultella planticola: a case report and literature review.’ Infection. 2015;43(5):621–2.

24. Yoon JH, Ahn YH, Chun JI, Park HJ, Park B-K. Acute Raoultella planticola cystitis in a child with rhabdomyosarcoma of the bladder neck. Pediatr Int. 2015;57(5):985–7.

25. Kim SW, Kim JE, Hong YA, Ko GJ, Pyo HJ, Kwon YJ. Raoultella planticola peritonitis in a patient on continuous ambulatory peritoneal dialysis. Infection. 2015;43(6):771–5.

26. Cho YJ, Jung EJ, Seong JS, Woo YM, Jeong BJ, Kang YM, et al. A Case of Pneumonia Caused by Raoultella planticola. Tuberc Respir Dis (Seoul). 2016;79(1):42–5.

27. Vassallo J, Vella M, Cassar R, Caruana P. Four cases of Raoultella planticola conjunctivitis. Eye. 2016 Apr 8;30(4):632–4.

28. Gian J, Cunha BA. Raoultella planticola chronic bacterial prostatitis with prostatic calcifications: successful treatment with prolonged fosfomycin therapy. Int J Antimicrob Agents. 2016;47(5):414.

29. de Campos FPF, Guimarães TB, Lovisolo SM. Fatal pancreatic pseudocyst co-infected by {Raoultella} planticola: an emerging pathogen. Autops Case Reports. 2016;6(2):27–31.

30. Sitaula S, Shahrrava A, Al Zoubi M, Malow J. The first case report of Raoultella planticola liver abscess. IDCases. 2016;5:69–71.

31. Papaefstathiou E, Gkekas C, Malioris A, Stafilarakis D, Papathanasiou M. Purple urinary bag syndrome due to raoultella ornitholytica, a rare presentation of a rare uropathogen. Eur Urol Suppl. 2016;15(10):e1335.

32. Pan Z, Liu R, Zhang P, Zhou H, Fu Y, Zhou J. Combination of Tigecycline and Levofloxacin for Successful Treatment of Nosocomial Pneumonia Caused by New Delhi Metallo-β-Lactamase-1-Producing Raoultella planticola. Microb Drug Resist. 2017;23(1):127–31.

33. Skelton WP, Taylor Z, Hsu J. A rare case of Raoultella planticola urinary tract infection in an immunocompromised patient with multiple myeloma. IDCases. 2017;8:9–11.

34. Westerveld D, Hussain J, Aljaafareh A, Ataya A. A Rare Case of Raoultella planticola Pneumonia: An Emerging Pathogen. Respir Med Case Reports. 2017;21:69–70.

35. Adjodah C, D’Ivernois C, Leyssene D, Berneau J-B, Hemery Y. A cardiac implantable device infection by Raoultella planticola in an immunocompromized patient. JMM Case Reports. 2017 Feb 28;4(2).

36. Tuğcu M, Ruhi C, Gokce AM, Kara M, Aksaray S. A case of urinary tract infection caused by Raoultella planticola after a urodynamic study. Braz J Infect Dis. 2017 Mar;21(2):196–8.

37. Howell C, Fakhoury J. A case of Raoultella planticola causing a urinary tract infection in a pediatric patient. Transl Pediatr. 2017;6(2):102–3.

38. Atıcı S, Alp Ünkar Z, Öcal Demir S, Akkoç G, Yakut N, Yılmaz Ş, et al. A rare and emerging pathogen: {Raoultella} planticola identification based on 16S {rRNA} in an infant. J Infect Public Health. 2017;

39. Bonnet E, Julia F, Giordano G, Lourtet-Hascoet J. Joint infection due to Raoultella planticola: first report. Infection. 2017;45(5):703–4.

40. Bardellini E, Amadori F, Schumacher RF, Foresti I, Majorana A. A new emerging oral infection: Raoultella planticola in a boy with haematological malignancy. Eur Arch Paediatr Dent. 2017 Jun 27;18(3):215–8.

41. Ulukent SC, Sarici İS, Alper Sahbaz N, Ozgun YM, Akca O, Sanlı K. Is It Necessary to Specifically Define the Cause of Surgically Treated Biliary Tract Infections? A Rare Case of Raoultella planticola Cholecystitis and Literature Review. Case Rep Infect Dis. 2017;2017:4181582.

42. Subedi R, Dean R, Li W, Dhamoon A. A novel case of Raoultella planticola osteomyelitis and epidural abscess. BMJ Case Rep. 2017 Jul 13;2017:bcr-2017-220329.

43. Povlow MR, Carrizosa J, Jones A. Raoultella Planticola: Bacteremia and Sepsis in a Patient with Cirrhosis. Cureus. 2017 Jul 24;9(7):e1508.

44. Yoshida N, Tsuchida Y. Palm atheroma infection caused by Raoultella planticola. BMJ Case Rep. 2017 Nov 4;2017:bcr-2017-222541.

45. Kalaria SS, Elliott K, Combs N, Phillips LG. Raoultella planticola: A Rare Cause of Wound Infection. Wounds a Compend Clin Res Pract. 2017 Nov;29(11):E103–5.

46. Yamamoto S, Nagatani K, Sato T, Ajima T, Minota S. Raoultella planticola Bacteremia in a Patient with Early Gastric Cancer. Intern Med. 2018 May 15;57(10):1469–73.

47. Naganathan G, Amin NK. Raoultella Planticola associated necrotizing appendicitis: A novel case report. Int J Surg Case Rep. 2018;44:38–41.

48. Lam PW, Tadros M, Fong IW. Mandibular osteomyelitis due to Raoultella species. JMM Case Reports. 2018 Mar 1;5(3).

49. Yumoto T, Naito H, Ihoriya H, Tsukahara K, Ota T, Watanabe T, et al. Raoultella planticola bacteremia-induced fatal septic shock following burn injury. Ann Clin Microbiol Antimicrob. 2018 Dec 4;17(1):19.

50. Mehmood H, Pervin N, Israr Ul Haq M, Kamal KR, Marwat A, Khan M. A Rare Case of Raoultella planticola Urinary Tract Infection in a Patient With Immunoglobulin A Nephropathy. J Investig Med high impact case reports. 2018 Jan 5;6:2324709618780422.

51. Gonzales Zamora JA, Corzo-Pedroza M, Romero Alvarez M, Martinez O V. Carbapenemase-Producing Raoultella Planticola: A Rare Cause of Pneumonia and Bacteremia. Dis (Basel, Switzerland). 2018 Oct 17;6(4):94.

52. AlSweed A, Alghamdi A, Tufenkeji H, Al-Hajjar S. The first case of Raoultella planticola infective endocarditis in a 4 year old child: A case report and review of literature. Int J Pediatr Adolesc Med. 2018 Mar;5(1):28–30.

53. Fager C, Yurteri-Kaplan L. Urinary tract infection with rare pathogen Raoultella Planticola: A post-operative case and review. Urol Case Reports. 2019 Jan;22:76–9.

54. Al-Sawaf O, Garcia-Borrega J, Vehreschild JJ, Thelen P, Fätkenheuer G, Shimabukuro-Vornhagen A, et al. Pelvic cellulitis caused by Raoultella planticola in a neutropenic patient. J Infect Chemother. 2019 Apr;25(4):298–301.

55. Olivier M, Madruga M, Carlan S. Raoultella planticola, an Emerging Pathogen. Infect Dis Clin Pract. 2019 May;1.

56. Pacilli M, Nataraja RM. Raoultella planticola associated with Meckel’s diverticulum perforation and peritonitis in a child: Case report and systematic review of the paediatric literature. Vol. 12, Journal of Infection and Public Health. Elsevier Ltd; 2019. p. 605–7.

57. Park SC, Wailan AM, Barry KE, Vegesana K, Carroll J, Mathers AJ, et al. Managing all the genotypic knowledge: Approach to a septic patient colonized by different Enterobacteriales with unique carbapenemases. Antimicrob Agents Chemother. 2019;63(8).

58. Asif S, Abughanimeh OK, Husainat NM, Numan L. Maxillary Osteomyelitis with an Incidental Diagnosis of Maxillary Diffuse Large B-Cell Lymphoma: A Case Report. Cureus. 2019 Jul 25;11(7).

59. Harmon SL, Nadeem I. Recurrent urinary tract infections caused by Raoultella planticola after kidney transplant. Transpl Infect Dis. 2019 Dec 1;21(6).

60. Tufa TB, Fuchs A, Feldt T, Galata DT, Mackenzie CR, Pfeffer K, et al. CTX-M-9 group ESBL-producing Raoultella planticola nosocomial infection: First report from sub-Saharan Africa. Ann Clin Microbiol Antimicrob. 2020 Aug 17;19(1).

61. Chen X, Guo S, Liu D, Zhong M. Neonatal septicemia caused by a rare pathogen: Raoultella planticola - A report of four cases. BMC Infect Dis. 2020 Sep 16;20(1).

62. Zhao Y, Tang N, Jia R, Hu J, Liu W, Sun Y, et al. Co-existence of the carbapenem resistance genes blaKPC-2 and blaNDM-1 in a Raoultella planticola isolate in China. Vol. 23, Journal of Global Antimicrobial Resistance. Elsevier Ltd; 2020. p. 327–8.

63. Ismair K, Abdeen Y. A Rare Case of Joint Infection due to Raoultella planticola. Surg J. 2020 Oct;06(04):e185–7.

64. Yilmaz U, Kizilates F. A rare case of raoultella planticola peritonitis in a chronic ambulatory peritoneal dialysis patient and review of the literature. Niger J Clin Pract. 2021 Jan 1;24(1):132–4.

65. Blihar D, Phuu P, Kotelnikova S, Johnson E. Bacteremic cholangitis due to Raoultella planticola complicating intrahepatic bile duct stricture 5 years post-laparoscopic cholecystectomy: a case report. J Med Case Rep. 2021 Dec 1;15(1).

66. Erwes T, Abrantes-Figueiredo J. A novel case of Raoultella bacteremia secondary to liver abscess formation following transarterial chemoembolization. IDCases. 2021 Jan 1;24.

67. Deeb LS, Bajaj J, Bhargava S, Alcid D, Pitchumoni CS. Acute suppuration of the pancreatic duct in a patient with tropical pancreatitis. Case Rep Gastroenterol. 2008;2(1):27–32.

68. Morais VP, Daporta MT, Bao AF, Campello MG, Andrés GQ. Enteric Fever-Like Syndrome Caused by Raoultella ornithinolytica (Klebsiella ornithinolytica). J Clin Microbiol. 2009;47(3):868–9.

69. Vos B, Laureys M. [Giant renal cyst as cause of colic obstruction]. Rev Med Brux. 2009;30(2):107–9.

70. Mau N, Ross LA. Raoultella ornithinolytica bacteremia in an infant with visceral heterotaxy. Pediatr Infect Dis J. 2010;29(5):477–8.

71. Solak Y, Gul EE, Atalay H, Genc N, Tonbul HZ. A rare human infection of Raoultella ornithinolytica in a diabetic foot lesion. Ann Saudi Med. 2011;31(1):93–4.

72. Sener D, Cokhras H, Camcioglu Y, Akcakaya N, Cakir E. Raoultella infection causing fever of unknown origin. Vol. 30, Pediatric Infectious Disease Journal. 2011. p. 1122–3.

73. Hadano Y, Tsukahara M, Ito K, Suzuki J, Kawamura I, Kurai H. Raoultella ornithinolytica bacteremia in cancer patients: report of three cases. Intern Med. 2012;51(22):3193–5.

74. Khajuria A, Praharaj AK, Grover N, Kumar M. First Report of blaNDM-1 in Raoultella ornithinolytica. Antimicrob Agents Chemother. 2013;57(2):1092–3.

75. García-Lozano T, Pascual Plá FJ, Aznar Oroval E. [Raoultella ornithinolytica in urinary tract infections. Clinical and microbiological study of a series of 4 oncologic patients]. Med Clin (Barc). 2013;141(3):138–9.

76. Sandal G, Ozen M. Fatal Raoultella ornithinolytica sepsis and purpura fulminans in a preterm newborn. Indian J Paediatr Dermatology. 2014;15(1):24.

77. Sibanda M. Primary peritonitis caused by Raoultella ornithinolytica in a 53‐year‐old man. JMM Case Reports. 2014;1(3).

78. Haruki Y, Hagiya H, Sakuma A, Murase T, Sugiyama T, Kondo S. Clinical characteristics of Raoultella ornithinolytica bacteremia: a case series and literature review. J Infect Chemother Off J Japan Soc Chemother. 2014;20(9):589–91.

79. Kaya S, Bayramoglu G, Sönmez M, Köksal I, Kaya S, Bayramoglu G, et al. Raoultella ornithinolytica causing fatal sepsis. Brazilian J Infect Dis. 2015;19(2):230–1.

80. Bhatt P, Tandel K, Das NK, Rathi KR. New Delhi metallo-β-lactamase producing extensively drug-resistant Raoultella ornithinolytica isolated from drain fluid following Whipple’s pancreaticoduodenectomy. Med J Armed Forces India. 2015 Dec 1;71(Suppl 2):S609–11.

81. Nakasone ES, Kaneshiro R, Min K, Tokeshi J. Emergence of Raoultella ornithinolytica on O‘ahu: A Case of Community-acquired R. ornithinolytica Urinary Tract Infection. Hawai’i J Med Public Heal. 2015;74(5):174–5.

82. Sękowska A, Dylewska K, Gospodarek E, Bogiel T. Catheter-related blood stream infection caused by Raoultella ornithinolytica. Folia Microbiol (Praha). 2015;60(6):493.

83. Zheng B, Zhang J, Ji J, Fang Y, Shen P, Ying C, et al. Emergence of Raoultella ornithinolytica coproducing IMP-4 and KPC-2 carbapenemases in China. Antimicrob Agents Chemother. 2015;59(11):7086–9.

84. Yamakawa K, Yamagishi Y, Miyata K, Shimomura Y, Iwata A, Hori T, et al. Bacteremia Caused by Raoultella ornithinolytica in Two Children. Pediatr Infect Dis J. 2016;35(4):452–3.

85. Venus K, Vaithilingam S, Bogoch II. Septic arthritis of the knee due to Raoultella ornithinolytica. Infection. 2016;44(5):691–2.

86. Seng P, Theron F, Honnorat E, Prost D, Fournier P-E, Stein A. Raoultella ornithinolytica: An unusual pathogen for prosthetic joint infection. IDCases. 2016;5:46–8.

87. Sueifan M, Moog V, Rau E, Eichenauer T. Sepsis durch Raoultella ornithinolytica bei einem immunkompetenten Patienten. Anaesthesist. 2016 Feb 26;65(2):129–33.

88. Singh M, Kaur I, Mundi DK, Kaur A. ENT infection caused by Raoultella ornithinolytica. Niger J Clin Pract. 2017;20(7):914.

89. Jellinge ME. Raoultella Ornithinolytica Diagnosed in a Neurointensive Patient. A Rare Case with Recovery without Antibiotics. J Crit care Med (Universitatea Med si Farm din Targu-Mures). 2017 Jul 26;3(3):120–2.

90. Levorova J, Machon V, Guha A, Foltan R. Septic arthritis of the temporomandibular joint caused by rare bacteria Raoultella ornithinolytica. Int J Oral Maxillofac Surg. 2017 Jan 1;46(1):111–5.

91. Abbas A, Ahmad I. First report of neonatal early-onset sepsis caused by multi-drug-resistant Raoultella ornithinolytica. Infection. 2018 Apr 4;46(2):275–7.

92. González-Castro A, Rodríguez-Borregán JC, Campos S, Pérez Canga JL. Catheter-related bacteraemia caused by Raoultella ornithinolytica. Rev Esp Anestesiol Reanim. 2018 Feb;65(2):116–8.

93. Papakanderaki E, Kanakakis K, Goule S, Chounti M, Hountis P. Clinical significance of positive Raoultella Ornithinolytica and Staphylococcus hominis cultures in a post lobectomy patient. A case report. Monaldi Arch chest Dis = Arch Monaldi per le Mal del torace. 2018 Feb 26;88(1):885.

94. De Petris L, Ruffini E. Roultella ornithinolytica infection in infancy: a case of febrile urinary tract infection. CEN case reports. 2018 Nov 2;7(2):234–6.

95. Hajjar R, Schwenter F, Su S-H, Gasse M-C, Sebajang H. Community-acquired infection to Raoultella ornithinolytica presenting as appendicitis and shock in a healthy individual. J Surg case reports. 2018 May 1;2018(5):rjy097.

96. Ayoade F, Mada PK, Alam M. Fat necrosis and polymicrobial wound infection caused partly by Raoultella ornithinolytica after reduction mammoplasty. BMJ Case Rep. 2018 Jun 4;2018:bcr-2018-224234.

97. Van Cleve JR, Boucher BA, Smith D V, Croce MA. Ventilator associated pneumonia caused by Raoultella ornithinolytica in two immunocompetent trauma patients. Respir Med case reports. 2018;24:135–7.

98. Büyükcam A, Liste Ü, Bıçakçıgil A, Kara A, Sancak B. A case of Raoultella ornithinolytica urinary tract infection in a pediatric patient. J Infect Chemother. 2019 Jun;25(6):467–9.

99. Sánchez-Códez M, Lubián-Gutiérrez M, Blanca-García JA, Pérez Aragón C. Infección asociada a catéter porLeclercia adecarboxylata yRaoultella ornithinolytica en un paciente con enfermedad mitocondrial. Arch Argent Pediatr. 2019 Apr 1;117(2):e147–9.

100. Reyes JA, Villavicencio F, Villacís JE, Pavón E, Campoverde N, Espinel M, et al. First report of a clinical isolate of blaOXA-48- carbapenemase producing Raoultella ornithinolytica in South America. Vol. 52, Revista Argentina de Microbiologia. Asociacion Argentina de Microbiologia; 2020. p. 82–3.

101. Surani A, Slama EM, Thomas S, Ross RW, Cunningham SC. Raoultella ornithinolytica and Klebsiella oxytoca pyogenic liver abscess presenting as chronic cough. IDCases. 2020 Jan 1;20.

102. Cavaliere M, Bartoletti G, Capriglione P, Di Lullo AM, Motta G, Iengo M, et al. Unusual Localization of an Emergent Bacterium, Raoultella ornithinolytica. Case Rep Med. 2020;2020.

103. Pi DD, Zhou F, Bai K, Liu C, Xu F, Li J. Raoultella ornithinolytica Infection in the Pediatric Population: A Retrospective Study. Front Pediatr. 2020 Jul 10;8.

104. Prada-Avella MC, Luengas-Monroy MA, Suárez A, Mora-Ramírez LM, Faccini-Martínez ÁA. Raoultella ornithinolytica urinary tract infection in a pediatric patient with T-cell precursor acute lymphoblastic leukemia. Bol Med Hosp Infant Mex. 2021 Jun 10;

105. Goegele H, Ruttmann E, Aranda-Michel J, Kafka R, Stelzmueller I, Hausdorfer H, et al. Fatal endocarditis due to extended spectrum betalactamase producing Klebsiella terrigena in a liver transplant recipient. Wien Klin Wochenschr. 2007;119(11–12):385–6.

106. Shaikh MM, Morgan M. Sepsis caused by Raoultella terrigena. JRSM Short Rep. 2011;2(6):49.

107. Demiray T, Köroğlu M, Özbek A, Hafizoğlu T, Altındiş M. The first case of Raoultella terrigena infection in an infant. Turk J Pediatr. 2015;57(6):624–8.

108. Wang Y, Jiang X, Xu Z, Ying C, Yu W, Xiao Y. Identification of Raoultella terrigena as a Rare Causative Agent of Subungual Abscess Based on 16S rRNA and Housekeeping Gene Sequencing. Can J Infect Dis Med Microbiol = J Can Des Mal Infect La Microbiol Medicale. 2016;2016:3879635.

109. Mal PB, Sarfaraz S, Herekar F, Ambreen R. Clinical manifestation and outcomes of multi-drug resistant (MDR) Raoultella terrigena infection – A case series at Indus Health Network, Karachi, Pakistan. IDCases. 2019 Jan 1;18.

110. Lekhniuk N, Fesenko U, Pidhirnyi Y, Sękowska A, Korniychuk O, Konechnyi Y. Raoultella terrigena: Current state of knowledge, after two recently identified clinical cases in Eastern Europe. Clin Case Reports. 2021 May 1;9(5):4089.

111. Mori M, Ohta M, Agata N, Kido N, Arakawa Y, Ito H, et al. Identification of species and capsular types of Klebsiella clinical isolates, with special reference to Klebsiella planticola. Microbiol Immunol. 1989;33(11):887–95.

112. Podschun R, Ullmann U. Isolation of Klebsiella terrigena from clinical specimens. Eur J Clin Microbiol Infect Dis. 1992 Apr;11(4):349–52.

113. Chun S, Yun JW, Huh HJ, Lee NY. Clinical characteristics of Raoultella ornithinolytica bacteremia. Infection. 2015;43(1):59–64.

114. Seng P, Boushab BM, Romain F, Gouriet F, Bruder N, Martin C, et al. Emerging role of Raoultella ornithinolytica in human infections: a series of cases and review of the literature. Int J Infect Dis. 2016;45:65–71.

115. Al-Hulu SM, Al-Charrakh AH. Isolation and characterization of Raoultella ornithinolytica from Clinical Specimens in Hilla city, Iraq. Iraq Med J Babylon. 2009;(7):42–7.

116. Park JS, Hong KH, Lee HJ, Choi SH, Song SH, Song K-H, et al. Evaluation of three phenotypic identification systems for clinical isolates of Raoultella ornithinolytica. J Med Microbiol. 2011;60(Pt 4):492–9.

117. de Jong E, de Jong AS, Smidts-van den Berg N, Rentenaar RJ. Differentiation of Raoultella ornithinolytica/planticola and Klebsiella oxytoca clinical isolates by matrix-assisted laser desorption/ionization-time of flight mass spectrometry. Diagn Microbiol Infect Dis. 2013;75(4):431–3.

118. Chun S, Yun JW, Huh HJ, Lee NY. Low virulence? Clinical characteristics of Raoultella planticola bacteremia. Infection. 2014;42(5):899–904.

119. Boattini M, Almeida A, Cardoso C, Cruz CS, Machado C, Vesza Z, et al. Infections on the rise: Raoultella spp., clinical and microbiological findings from a retrospective study, 2010-2014. Infect Dis (London, England). 2016;48(1):87–91.

120. Ponce-Alonso M, Rodríguez-Rojas L, del Campo R, Cantón R, Morosini M-I. Comparison of different methods for identification of species of the genus Raoultella: report of 11 cases of Raoultella causing bacteraemia and literature review. Clin Microbiol Infect. 2016;22(3):252–7.

121. Demiray T, Koroglu M, Ozbek A, Altindis M. A rare cause of infection, Raoultella planticola: emerging threat and new reservoir for carbapenem resistance. Infection. 2016;44(6):713–7.

122. Hong G, Yong HJ, Lee D, Kim DH, Kim YS, Park J-S, et al. Clinical characteristics and treatment outcomes of patients with pneumonia caused by Raoultella planticola. J Thorac Dis. 2020 Apr 1;12(4):1305–11.

123. Ahmed I, Nasir N, Ali BJ, Mahmood SF. 1417. Clinical Features and Outcomes of raoultella terrigena Infections. A Single-Center Experience from Karachi, Pakistan . Open Forum Infect Dis. 2020 Dec 31;7(Supplement_1):S715–S715.

124. Mohammed Y, Gadzama GB, Zailani SB, Aboderin AO. Characterization of extended-spectrum beta-lactamase from Escherichia coli and Klebsiella species from North Eastern Nigeria. J Clin Diagnostic Res. 2016 Feb 1;10(2):DC07-DC10.

125. Alampoondi Venkataramanan SV, George L, Sahu KK, Abraham GM. A 5-Year Retrospective Analysis of Raoultella planticola Bacteriuria. Infect Drug Resist. 2021 May;Volume 14:1989–2001.

126. Seifu WD, Gebissa AD. Prevalence and antibiotic susceptibility of Uropathogens from cases of urinary tract infections (UTI) in Shashemene referral hospital, Ethiopia. BMC Infect Dis. 2018 Dec 10;18(1):30.

127. Bueno F, Huntley D, Navarro D, Colomina J. Antibiotic susceptibility of Raoultella spp. in the Valencian Community: Results of Microbiological Surveillance Network for 4 years. Rev Española Anestesiol y Reanim (English Ed. 2021 Mar 1;68(3):171–2.

128. Sêkowska A, Bogiel T, Woźniak M, Gospodarek-Komkowska E. Raoultella spp. – reliable identification, susceptibility to antimicrobials and antibiotic resistance mechanisms. J Med Microbiol. 2020 Feb 1;69(2):233–8.

129. Podschun R, Acktun H, Okpara J, Linderkamp O, Ullmann U, Borneff-Lipp M. Isolation of Klebsiella planticola from Newborns in a Neonatal Ward. J Clin Microbiol. 1998;36(8):2331–2.

130. Podschun R, Fischer A, Ullmann U. Characterization of Klebsiella terrigena strains from humans: haemagglutinins, serum resistance, siderophore synthesis, and serotypes. Epidemiol Infect. 2000;125(1):71–8.

131. Abid IN. Emergence of Raoultella ornithinolytica producing AmpC -Beta lactamases in the different clinical specimens. J Nat Sci Res. 2016;6(8):124–9.

132. Arteta AA, Carvajal-Restrepo H, Sánchez-Jiménez MM, Diaz-Rodriguez S, Cardona-Castro N. Gallbladder microbiota variability in Colombian gallstones patients. J Infect Dev Ctries. 2017;11(3):255–60.
